# Voluntary acceptance and consumption of two oral ciclosporin formulations in dogs: two randomised, controlled studies

**DOI:** 10.1186/s13620-015-0031-8

**Published:** 2015-02-12

**Authors:** Christelle Navarro, Nolwenn Crastes, Elodie Benizeau, David McGahie

**Affiliations:** Medical department, Virbac, 13ème rue LID, 06515 Carros, France; R&D, Virbac, 13ème rue LID, 06515 Carros, France

**Keywords:** Atopic dermatitis, Dog, Ciclosporin, Intake, Prehension, Voluntary acceptance, Consumption, Compliance

## Abstract

**Background:**

Atopic dermatitis (AD) is the most common canine allergic skin disease and can significantly affect the quality of life of affected dogs. Treating canine AD with ciclosporin has been a subject of great interest in recent years. Many studies have provided substantial evidence of ciclosporin efficacy and safety in canine AD management, and for several years ciclosporin has been recognised as a major component of canine AD multimodal therapy. As a chronic condition, canine AD requires life-long medical management and treatment success relies in large part on product ease of administration. Two studies were conducted to assess the comparative voluntary acceptance and consumption of Cyclavance® (Virbac), a new oral liquid formulation of ciclosporin, and Atopica® (Novartis) either added to a small quantity of kibbles (study 1) or administered directly into the dog’s mouth (study 2).

**Results:**

Over the course of the two studies 70 dogs assessed each of the ciclosporin formulations and 320 individual tests were performed for each tested product. Immediate prehension (in less than 2 seconds) occurred significantly more often with Cyclavance® (90.6% of the tests) than with Atopica® (14.4% of the tests) when products were mixed with 30 grams of dry food (p < 0.001). Moreover, Cyclavance® was significantly more often easily accepted than Atopica® (99.3% vs 27.1% of the tests, respectively) when products were administered directly into the dogs’ mouth (p < 0.0001). Cyclavance® was also more often totally consumed (98.3% of the tests) than Atopica® (2.2% of the tests) when mixed with a small amount of food (p < 0.001). However, both products were totally consumed once administered directly into the dogs’ mouth.

**Conclusions:**

By facilitating cicloporin administration and consumption, Cyclavance® liquid formulation offers an interesting alternative to capsules that may improve dosing compliance and therefore the ability to benefit from the therapeutic effects in the long-term treatment of canine AD.

## Background

Ciclosporin is a lipophilic cyclic polypeptide with powerful immunosuppressive and immunomodulatory properties which was first licensed for the management of canine atopic dermatitis (AD) in 2002 as Atopica® (Novartis Animal Health) and is now approved and available in over 20 countries worldwide.

Ciclosporin at an initial dosage of 5 mg/kg, PO, every 24 hours has been largely demonstrated to be highly effective in the treatment of AD in dogs and remains nowadays one of the most important tools within a multimodal treatment strategy [1]. As a consequence, the International Task Force for Canine Atopic Dermatitis (now the International Committee for Allergic Diseases in Animals [ICADA]) practice guidelines for the treatment of canine AD has specifically been recommending the use of ciclosporin as one of the management tool for chronic AD since 2010. However, the non-palatable capsule formulation of Atopica raises some concerns about compliance in a long-term treatment strategy.

A new oral liquid formulation of ciclosporin (Cyclavance®, Virbac) has recently been approved in Europe, thus offering an alternative method of administration and making it possible to adjust individual dosages with accuracy. It is a 100 mg/ml oral solution indicated in dogs for the treatment of chronic clinical manifestations of AD. After oral administration of ciclosporin at the labeled recommended dose rate of 5 mg/kg bodyweight, peak blood concentrations are achieved within 1–2 h [2]. Being lipophilic, ciclosporin is distributed widely in the body with skin and adipose tissue acting as principal storage sites. The concentration of ciclosprin in skin is up to 10 times higher than that in blood. The high skin concentrations and the relatively long half-life of the drug (average 8.6 h) support once daily dosing in dogs [2,3]. Therefore, Cyclavance®, like Atopica®, could initially be given daily until a satisfactory clinical improvement is seen and then can be given every other day, and eventually in some cases every 3 to 4 days, as a maintenance dose [4].

The liquid form of ciclosporin has been designed to be easier to administer than the capsule form. Our hypothesis was therefore that it would be more acceptable to dogs and reduce the need for forced administration. It was decided to compare, in two separate studies, the dogs’ voluntary acceptance and consumption of both presentations when presented in a bowl mixed with a small amount of food (study 1), and when administered directly into the mouth (study 2).

## Methods

### Tested products

The products tested were Cyclavance® 100 mg/ml oral solution (Virbac, France) and Atopica® 50 mg or Atopica® 100 mg capsules (Novartis Animal Health, Switzerland). The products were administered orally.

### Ethics statement

The studies were carried out in accordance with the relevant European legislation and Virbac’s chart of Ethics (Study 1: EU-2014/11-13; Study 2: CE VB-2012-25-001).

### Study 1

#### Animals

Sixty adult healthy dogs, 32 females and 28 males, were included. The breeds represented included various unspecified mixed breeds (58%), Beagle (20%), Fox Terrier (8%), Spaniel (5%), Cocker Spaniel (3%), Jack Russel Terrier, Shetland Sheepdog, and Schnauzer (less than 2% each). The dogs were aged between 1.5 and 16.2 years with a mean age of 7.3 (±3.3) years and weighed between 7.7 and 23.8 kg with a mean weight of 13.6 (±4.0) kg. Animals were housed, managed and fed as normal and no alteration was required for the purpose of the study. Water was given *ad libitum*. The dogs were fed once per day with a complete dry maintenance food for adult dogs at 8 am each day.

### Study design

This mono-center, controlled, randomised cross-over study was conducted in accordance with the rules of the European Union on Animal welfare in a specialised kennel. Given the obvious difference between the formulations (oral solution vs capsule) of the two tested products, the palatability tests could not be blinded. At enrolment the animals were divided into three groups consisting of twenty dogs each according to their bodyweight (≤10 kg, 10–14.9 kg, ≥15 kg). Dogs were then randomly allocated within each group to Sequence A or Sequence B in a tiered fashion based on their sex and age. The testing consisted of two sequences of three days of product administration separated by two days of wash-out. Sequence A consisted of Atopica® for three days, followed by two days of wash-out, then Cyclavance® for three days. Sequence B consisted of Cyclavance® for three days, followed by two days of wash-out, then Atopica® for three days.

### Product testing

The prehension and consumption of Cyclavance® was compared with that of Atopica® by the means of acceptance tests. These started at 2 pm on each study day. The dogs received 0.05 ml/kg of Cyclavance® or one capsule of Atopica® mixed with 30 grams of dry food presented in a bowl (Cyclavance® was poured onto the kibbles and the whole capsule of Atopica® was buried in kibbles). According to label recommendation, the dogs weighing between 7.5 and 15 kg were administered Atopica® 50 mg and the dogs weighing between 15 and 29 kg were presented with Atopica® 100 mg. The bowl containing the food and product was positioned on the floor inside the animal’s pen. A timer was started when the dog was allowed access to the bowl and was stopped when the product entered the animal’s mouth. The time to take the product was recorded as less than 2 seconds or more than 2 seconds. If after 60 seconds, the product had not been taken into the mouth, the test was terminated and the prehension was assessed as “no intake”. Each time kibbles or the capsule were prehended, the dog was observed during a further 5 minutes in order to register if it swallowed the product or if it spat it out. The amount of liquid and food remaining in the bowl were also recorded.

### Classification of product prehension and consumption

Prehension was defined as the animal voluntarily taking the product into the mouth. The prehension of the tested products was further classified as immediate (less than 2 seconds), delayed (more than 2 seconds), or no prehension. Product and kibble consumption was classified as total (100%), partial, and none (0%).

### Tolerance assessment

The dogs were monitored closely throughout the study with a requirement for any abnormal events to be recorded in the individual clinical report form of each dog.

### Experiment 2

#### Animals

Twenty adult healthy Beagle dogs, 12 females and 8 males, were enrolled. The dogs were aged between 0.5 and 3.2 years with a mean age of 2.2 (±0.8) years and weighed between 9.6 and 14.2 kg with a mean weight of 11.6 (±1.5) kg. Water was given *ad libitum*. The dogs were fed once per day with a complete dry maintenance food for adult dogs and housed in individual pens.

### Study design and test procedure

For this mono-center, controlled, randomised study the animals were randomly allocated to receive Cyclavance® or Atopica® in a tiered fashion according to their bodyweight and sex. The products were administered once per day during the 14-day study period at least 2 hours before meal time. In the Cyclavance® group the dosage was progressively increased from 1 to 4 mg/kg over a four-day period (from D0 to D3) then 5 mg/kg were administered daily over the ten remaining days (from D4 to D13). In the Atopica® group one capsule of Atopica® 50 mg was administered daily to all dogs as per the label recommendations. As a result, the dosage of ciclosporin given in this group varied between 3.5 and 5.2 mg/kg depending on bodyweight.

### Classification of product voluntary acceptance and consumption

Cyclavance® was administered directly in the dog’s mouth with the syringe. Atopica® capsules were offered by the hand and if not taken within 1 minute were inserted into the mouth.

The product intake was classified as voluntary acceptance (Syringe easily inserted into the mouth or capsule taken from the hand of the technician, combined with willingness to swallow the product) or forced administration (need for a strong animal handling to insert the syringe into the dog’s mouth or need to insert the capsule at the back of the throat or need for restraint to ensure swallowing).

### Tolerance assessment

A clinical examination of each dog including stool appearance, appetite and overall condition was performed before the beginning of the test procedure (on D0) and on each study day. Photographs of the faecal matter were taken each morning. Fecal scores were then blindly assessed at study completion by two different operators for each dog on each study day using a previously-validated scoring chart (scores 0–5) [5]. Furthermore, all dogs were weighed on D0, D7 and D14 and fasting blood samples were taken on D0 and D14 for haematology and biochemistry analysis. The number of dogs showing at least one gastrointestinal event (vomiting and/or diarrhoea) occurring over the first 4 study days and during the course of the entire study was compared between product groups.

### Statistical analyses

Statistical analyses were performed using SAS 9.3. In study 1, product prehension, product consumption and food consumption were compared between products using a general linear model (Proc GLM in SAS) for crossover study, with sequence, dog (sequence), product, period and product*period as fixed effects. This model was used to show if there was a relation or not between the product prehension, the product consumption, the food consumption, and the product, taking into account the period and the sequence.

In study 2, product acceptance was compared between products using a generalized linear model for repeated measures (Proc GENMOD in SAS), with group and day as fixed effects. This model was used to show if there was a relation or not between the product acceptance and the product, taking into account the repeatability of the measures (once per day, during 14 days).

The significance threshold was set at 5% for both studies.

## Results

### Study 1

**Figure 1:**
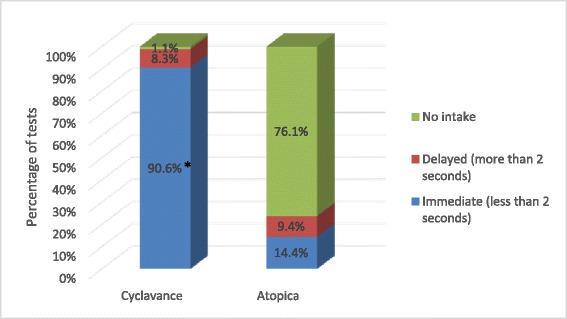
**Product prehension in study 1.** This chart presents the level of spontaneous prehension for the tested products. * Statistical difference between the products (p < 0.05). Immediate prehension rates were significantly higher for Cyclavance® compared to Atopica® (p < 0.001).

**Figure 2:**
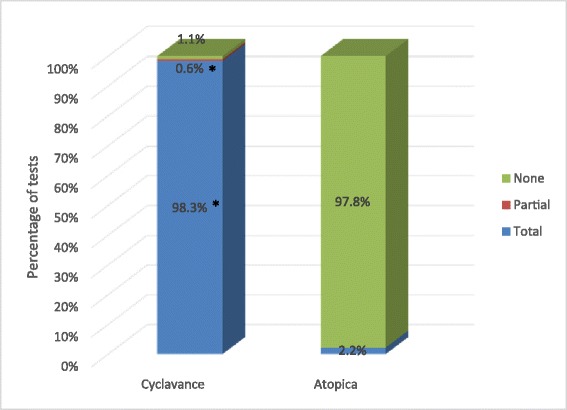
**Product consumption in study 1.** This chart presents the level of consumption for the tested products. * Statistical difference between the products (p < 0.05). Total consumption was significantly higher for Cyclavance® than Atopica® (p < 0.001).

Over the course of the study, a total of 180 individual voluntary acceptance tests were completed for each tested product. Figure 1 demonstrates the prehension by category for each product. Immediate prehension rates (product voluntarily taken into the dog’s mouth in less than 2 seconds) were significantly higher for Cyclavance® (163 of 180 tests; 90.6%) compared to Atopica® (26 of 180 tests; 14.4%) (p < 0.001). Table 1 shows the breakdown of prehension rates for each tested product by bodyweight range. The results remain in line with those observed with all dogs independently of their bodyweight. When considering product consumption, a statistically significant difference (p < 0.001) between products was also shown in favour of Cyclavance® with total consumption in 177 tests (98.3%) versus 4 tests (2.2%) for Atopica®. Figure 2 provides the details of the consumption rates by category. In one test (0.6%), a dog consumed partially the dose of Cyclavance®, but in all other tests, the dogs ingested the total amount of liquid with the kibbles. Some dogs took the capsule of Atopica® into their mouth and then spat it out immediately, but the great majority of the dogs did not take the capsule. The dogs’ bodyweight ranges had no impact on consumption for either product as shown in Table 2. Kibbles were totally (100%) consumed for both products. No adverse event was reported during the course of the study for either tested product.

**Table 1:** **Breakdown of product prehension rates per dogs’ bodyweight range in study 1**

**Dogs’ weight**	**Product**	**Prehension**		
		**Immediate (less than 2 seconds)**	**Delayed (more than 2 seconds)**	**No intake**
Less than 10 kg	**Cyclavance®**	54 (90%)	6 (10%)	0 (0%)
	**Atopica®**	15 (25%)	6 (10%)	39 (65%)
10 to 15 kg	**Cyclavance®**	60 (100%)	0 (0%)	0 (0%)
	**Atopica®**	7 (11.7%)	6 (10%)	47 (78.3%)
More than 15 kg	**Cyclavance®**	49 (81.7%)	9 (15%)	2 (3.3%)
	**Atopica®**	4 (6.7%)	5 (8.3%)	51 (85%)

**Table 2:** **Breakdown of product consumption per dogs’ bodyweight range in study 1**

**Dogs’ weight**	**Product**	**Consumption**		
		**Total**	**Partial**	**None**
Less than 10 kg	**Cyclavance®**	60 (100%)	0 (0%)	0 (0%)
	**Atopica®**	2 (3.3%)	0 (0%)	58 (96.7%)
10 to 15 kg	**Cyclavance®**	60 (100%)	0 (0%)	0 (0%)
	**Atopica®**	2 (3.3%)	0 (0%)	58 (96.7%)
More than 15 kg	**Cyclavance®**	57 (95%)	1 (1.7%)	2 (3.3%)
	**Atopica®**	0 (0%)	0 (0%)	60 (100%)

### Study 2

**Figure 3:**
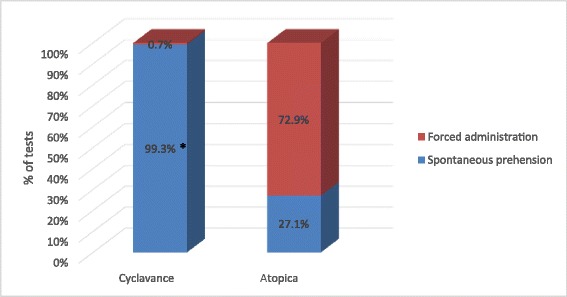
**Overall product voluntary acceptance in study 2.** This chart presents the voluntary acceptance of the tested products (all tests combined). * Statistical difference between the products (p < 0.05). Cyclavance® was significantly more often spontaneously prehended than Atopica® (p < 0.0001).

**Figure 4:**
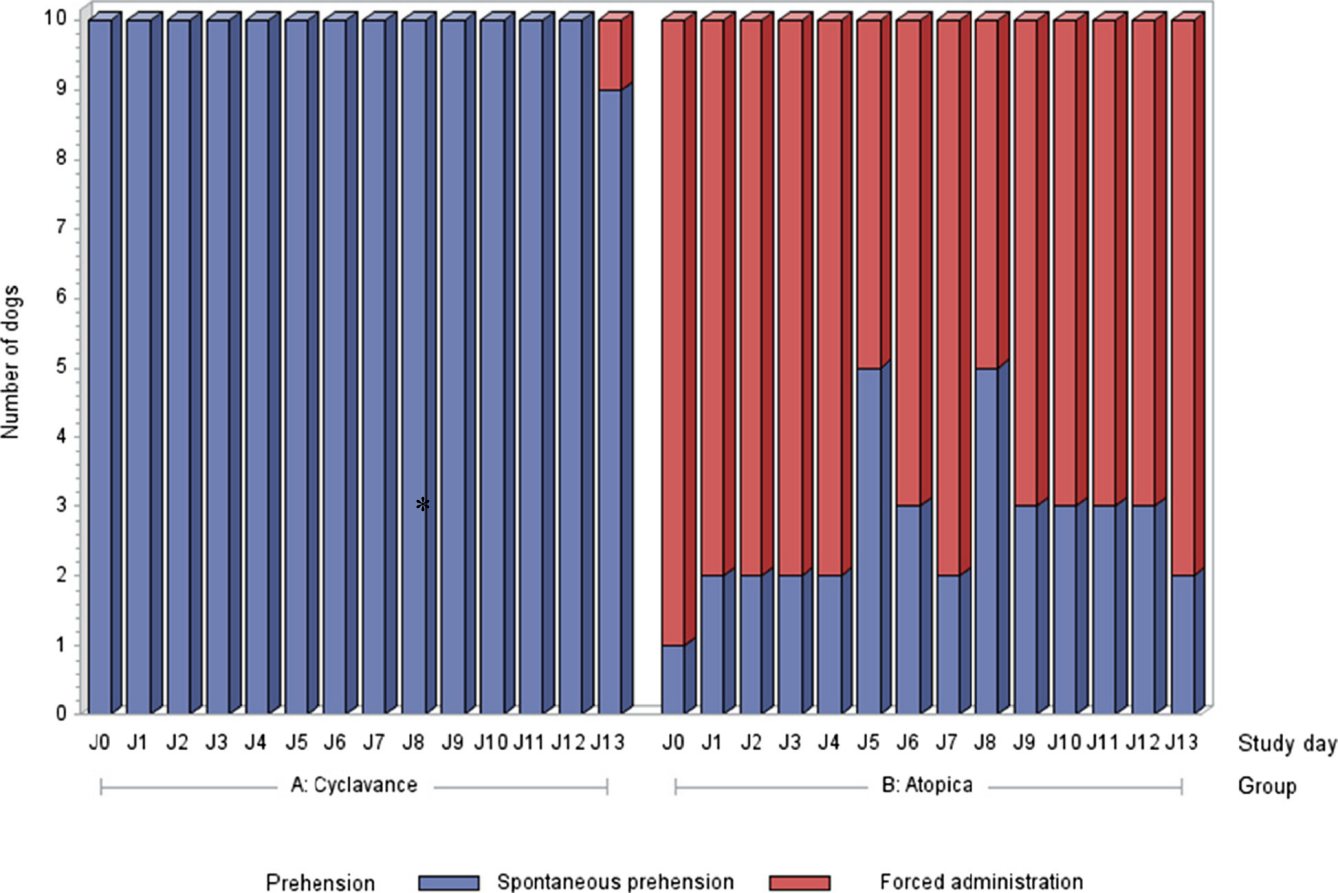
**Evolution of product voluntary acceptance throughout the study 2 period.** This chart presents the voluntary acceptance of the tested products on each study day. * Statistical difference between the products (p < 0.05). Cyclavance® was significantly more often easily accepted than Atopica® throughout the study period without any influence of study days (p < 0.0001).

Over the study period, a total of 140 tests were performed for each tested product. Cyclavance® was significantly more often easily accepted than Atopica® (99.3% vs 27.1% respectively; p < 0.0001) as shown in Figure 3. When focusing on the study period between D4 and D13 where all dogs received approximately 5 mg/kg ciclosporin daily, a similar difference between groups was noted (99.0% vs 31.0% respectively; p < 0.0001). Statistical analysis further confirmed that the same observation remained valid throughout the study period without any influence of study days as shown in Figure 4. In most tests, Atopica® had to be forcibly administered whereas Cyclavance® was easily accepted by the dogs in 100% of the tests on all study days except on D13 for one dog. One animal given Atopica® from D0 to D13 was able to spit out the capsule even when it was inserted into the back of the throat. Nevertheless, with forced administration it was always possible to ensure that the product was totally consumed except on 2 occasions where a dog bit the soft Atopica® capsule and the contents were partially spilt on the floor. No significant changes were noted in the dogs’ bodyweight. Rectal temperatures remained within normal values for all dogs. The overall condition was good for all dogs throughout the study period. No dog showed dehydration or a change of appetite. Fecal scores were very similar between both products. However, some dogs showed transitory diarrhoea and/or vomiting throughout the study period. During the 14-day study duration, there was no difference in the number of animals showing at least one gastro-intestinal event (6 in the Atopica group vs 7 in the Cyclavance group). However, over the first 4 days of the study, during which the Cyclavance group was receiving a progressively increasing dose, there were half as many dogs showing at least one gastrointestinal event in the Cyclavance group versus the Atopica group (respectively 3 vs 6). Haematological and biochemical parameters remained within normal ranges between D0 and D14 in all dogs.

## Discussion

Canine AD is a chronic disease that requires long-term treatment strategy. Thus, ease of administration, voluntary acceptance and consumption of drugs used for AD management are of primary importance to promote treatment success. To the authors’ knowledge, despite a high number of publications and studies performed on dogs, up to now no study had been specifically designed to investigate the acceptance and the consumption of ciclosporin. The procedures used in the reported studies (appropriate investigator and animal training to test procedures, tests performed at a fixed time every day, use of a cross-over design and of a randomised allocation plan) are derived from those used in the pet food industry in order to reduce bias [6,7]. Furthermore, 20 dogs and at least two days were reported in previous publications to be sufficient when determining palatability differences between any two food samples with sufficient accuracy and efficiency [8]. In the present studies, acceptance tests were run over a period of as long as 14 days. However, some limitations of the reported studies can be pointed out. As for most palatability studies, study 2 was performed with only one dog breed (Beagles) and even if no breed recommendations have been made for assessing both food and drug palatability, the sample used was not representative of the real population of dogs. The environmental conditions were not strictly controlled throughout study 1 and it is not possible to discount the possibility that variations may have affected the result between testing periods. Finally, it has been suggested that acceptance of a product may differ between animals kept under experimental and field conditions and that definitive assessment of palatability should be done in the consumers' home [9]. However, the results of the two studies reported in this paper certainly give clear indications on how Cyclavance® will be accepted by owners’ dogs. Indeed, since the assessment of its full consumption can be considered as a direct measure of its compliance [9], Cyclavance®, either administered with a small amount of food or directly into the dogs’ mouth, should help facilitate long-term administration. In that sense, it is interesting to note that, in study 1, 59 dogs out of 60 had a complete consumption of the 3 doses of Cyclavance® over the 3-day study period (indeed only 1 dog had partial consumption on day 1 and then no consumption on days 2 and 3) whereas no dogs in the Atopica group took the 3 doses (2 dogs took 2 doses). Furthermore, besides the ease of use of an oral syringe, Cyclavance® also enables more accurate dosage.

In an attempt to further facilitate product administration, ciclosporin was administered with a small amount of food in study 1. Knowing that the average food consumption per dog and per day was comprised between 200 and 400 grams during the course of this study, the 30 grams of kibbles given with the products represented about 10% of the dogs’ daily food intake. Moreover, considering that the acceptance tests took place each day 5.5 hours after the dogs’ meal time, it can be assumed that in study 1, the study design is compatible with the recommendation that the products should be given apart from meals. The effect of feeding on ciclosporin oral bioavailibity and efficacy is controversial in the literature. The absolute oral bioavailability of ciclosporin in dogs is low and highly variable. This can be explained by the high molecular weight of the drug, its low water solubility, the effect of the P-glycoprotein efflux pump at the intestinal level, and metabolism by cytochrome P450 3A enzymes located in the small intestinal mucosa and liver [2]. The product labelling for Atopica® and Cyclavance® therefore mention that both products should be administered at least 2 hours apart from feeding rather than at mealtimes. This precaution of use is supported by one study that tested the influence of the feeding status at the time of administration. The administration of ciclosporin to healthy beagles with their daily meal decreased the bioavailability and increased the individual variability of drug absorption [10]. In the same study, pharmacokinetic parameters in fed or fasted dogs receiving ciclosporin as solution or capsule were measured. Cmax tended to be lower after capsule administration and was also decreased by 23% in fed dogs compared with fasted dogs. In five of the 16 dogs which received the drug with food, Cmax and AUC did not reach 50% of the mean values obtained in the fasted dogs [10]. However, clinical response to therapy is a more reliable method of assessing efficacy of ciclosporin than interpretation of ciclosporin blood level. Moroever no correlation was found between clinical improvement and ciclosporin blood concentrations [11]. Indeed, in AD, ciclosporin target cells include Langerhans cells, mast cells, eosinophils and keratinocytes located in the skin. Since the drug concentration in epidermis and dermis is about 10-fold higher than in the blood, drug concentration in the skin may be sufficient to inhibit the activation of these cells involved in the inflammatory process [10]. Furthermore, another study found that administration of ciclosporin with food to dogs treated for AD did not influence the clinical response [12] and clinical experience has shown that efficacy seems unaffected by administration with food [3]. In conclusion, mixing ciclosporin with a small amount of food (i.e. 30 grams as in study 1) for easier administration should have, if any, very limited effects on its bioavailability or, more importantly, on the clinical response when used in canine AD.

Results of previous studies have suggested that ciclosporin is safe in dogs. Most adverse effects are manageable and/or without clinical significance, even over many years of treatment [1]. The most common adverse effects are gastrointestinal tract abnormalities during the first weeks of treatment [13,14]. In a meta-analysis of 672 atopic dogs treated with ciclosporin, gastrointestinal problems were recorded in 45 percent of dogs [15]. Vomiting was reported in 25 to 31 % of dogs and soft stools, diarrhea and/or other problems affected 18 to 20 % of the dogs receiving ciclosporin [15]. Most of the gastrointestinal upsets appear to be mild, require medical intervention only in rare instances [14], rarely require discontinuation of ciclosporin [14], and generally improve spontaneously upon further administration [16]. When drug related, most of these reactions appear to be dose dependent, and they resolve with dose reduction or treatment discontinuation [5]. Suggestions from the literature to reduce the incidence of vomiting and/or diarrhoea are to administer ciclosporin with a small amount of food [11] and/or to start treatment with a low dose (1 to 2 mg/kg every 24 hours) gradually increasing to the therapeutic dose (5 mg/kg every 24 hours) [15]. In study 2 we took advantage of the flexibility permitted by the use of the oral dosing syringe with a liquid formulation and applied this strategy for Cyclavance®. However the number of dogs in this study was not sufficient to answer the question of whether there will be any statistically significant difference between dogs receiving a progressive dosage increase of ciclosporin from 1 to 5 mg/kg over the first five days of dosing compared to those administered 5 mg/kg bodyweight from the onset of treatment. This subject should be further explored in future studies in field conditions using atopic dogs.

Such a progressive onset of treatment is unlikely to have any real impact on treatment efficacy. Usually, even if owners report a significant reduction of pruritus as early as 7–8 days in dogs treated with ciclosporin alone, it requires approximately three to four weeks of therapy at 5 mg/kg once daily for maximal clinical improvement [3,16]. The extent and severity of skin lesions is improved by approximately 50% in the first 4 weeks of treatment with a further improvement of up to 60–80% of the lesions severity obtained in the maintenance phase. The extent of improvement seems to be however more dependent upon treatment duration than the daily dose related to the body weight [10]. Furthermore, in a study where the initial dosage of ciclosporin ranged from 3.3 to 6.6 mg/kg, PO, every 24 hours, it was found that in this dosage range, there was no relationship between dosage and clinical response after 4 weeks [10]. This suggests that in this study, clinical response plateaued at dosages higher than 3.3 mg/kg. On the other hand, in a previous study a dosage of 2.5 mg/kg, PO, every 24 hours for 6 weeks was found to be significantly less effective than a dosage of 5 mg/kg [14]. These data suggest the need to conduct studies to evaluate ciclosporin efficacy in the treatment of canine AD after progressive dosage increase during treatment initiation and to investigate the impact on the frequency of side effects observed during this period.

## Conclusion

The liquid formulation of Cyclavance® is easy to administer and enables accurate dosage adjustments. Furthermore, the results of two distinct studies showed that the voluntary prehension, acceptance and consumption of Cyclavance® is significantly better than that of Atopica® when administered either directly into the dog’s mouth or mixed with a small amount of food. In chronic diseases such as canine AD requiring a long-term treatment strategy, these attributes are of primary importance for good patient and owner dosing compliance and thereby therapeutic success.

## Abbreviations

AD: Atopic dermatitis
AUC: Area under curve
